# Spirituality and quality of life among bipolar disorder patients

**DOI:** 10.1192/j.eurpsy.2021.530

**Published:** 2021-08-13

**Authors:** S. Ajmi, R. Sellami, S. Najjar, R. Masmoudi, I. Feki, J. Masmoudi

**Affiliations:** 1 Psychiatrie A, hospital University Hedi Chaker, sfax, Tunisia; 2 Psychiatrie “a” Department, Hedi Chaker Hospital University -Sfax - Tunisia, sfax, Tunisia; 3 Psychiatry A, hedi chaker hospital, Sfax, Tunisia

**Keywords:** Spirituality, quality of life, biopolar disorder

## Abstract

**Introduction:**

Quality of life is a broad and complex concept, but essentially refers to an individual’s well-being in a spectrum of life domains.

**Objectives:**

The aim of the present study was to investigate the relationship between spirituality, religiosity (S/R) and quality of life (QOL) among bipolar disorder (BD) patients.

**Methods:**

Data were collected between July and December 2017.Participants were enrolled from the Mood Consultation of the Psychiatry (A) Department of the University Hospital HediChaker. We assessed symptoms of mania [Young Mania Rating Scale (YMRS)], depression [Beck scale], quality of life [World Health Organization Quality of Life-Brief Version (WHOQOL-BREF)]andquality of life aspects related to spirituality, religiousness and personal beliefs [World Health Organization Quality Of Life –Spirituality, Religiousness and Personal Beliefs (WHOQOL-SRPB)]

**Results:**

Our sample included 60 patients. It consisted of 55% of female and the mean age was 44.94 (SD=12.76). The sample included 68% of participant diagnosed with BDI and 32% with BDII The median score of quality of live was 3 (minimum=1; maximum=5).The median of physic, psychic, social and environmental quality of life was respectively (25, 31, 81 and 19) (Minimum=0; Maximum=100). The mean score of WHOQOL-SPRB was 14.82 (Minimum=4, Maximum=20). S/R were correlated to psychic, social and environmental quality of life (p=0.006, p=0.011, p=0.016). We did not find a significant association between physic quality of life and S/R (p=0.234).

**Conclusions:**

Our study suggests that spirituality, religiosity have an important influence on most aspects of the quality of life among bipolar patients.

